# Value-Based Care and Anesthesiology in the USA

**DOI:** 10.7759/cureus.44410

**Published:** 2023-08-30

**Authors:** Faizan Ahmed, Bharath Chithrala, Kanchan Barve, Sara Biladeau, Sean P Clifford

**Affiliations:** 1 Anesthesiology, University of Louisville School of Medicine, Louisville, USA; 2 Anesthesiology, University of Pikeville, Pikeville, USA; 3 Health, Humana Inc., Louisville, USA; 4 Anesthesiology and Perioperative Medicine, University of Louisville School of Medicine, Louisville, USA

**Keywords:** value based care, quality improvement research, pulmonology and critical care, anesthesia efficacy, health quality

## Abstract

Value-based care, prioritizing patient outcomes over service volume, is steering a transformative course in anesthesiology in the United States. With the rise of this patient-centric approach, anesthesiologists are adopting dynamic roles to meet the demands of medical institutions, insurers, and patients for high-quality, cost-effective care. The urgency for this transition is accentuated by persistent challenges in reducing postoperative mortality rates and surgical complications, further spotlighted by the coronvirus disease 2019 (COVID-19) pandemic. Anesthesiologists engage in preoperative optimization, personalized care delivery, and evidence-based practices, bolstering their influence in the perioperative environment. Their collaboration with perioperative stakeholders propels the shift toward a value-driven healthcare landscape. This review analyzes the implementation of value-based care in American anesthesiology, assesses the significance of technology in enhancing its delivery, and outlines potential strategies for improving its application.

## Introduction and background

Healthcare spending in the United States skyrocketed to $3.2 trillion in 2015, comprising 17.8% of the nation's GDP. Fast-forward to 2020, predictions placed this figure at a staggering $4.6 trillion, or approximately 20% of the GDP [1]. A breakdown of the 2015 expenditure revealed that Medicare, Medicaid, and private health coverage consumed 20%, 17%, and 33% of the sum respectively [2]. Rising healthcare costs necessitate that nearly half of the government's medical benefit programs are currently sustained through means outside wage taxes and levies.

To address escalating costs and enhance the quality of patient care, the traditional fee-for-service (FFS) payment system, which primarily compensates providers based on the quantity of services rendered, has been replaced by value-based payment methods [3]. For instance, in the FFS model, a physician might be paid for each individual procedure they perform, regardless of the patient's outcome, emphasizing volume over value. This model has been criticized for potentially incentivizing unnecessary procedures and tests. Unforeseen consequences of the coronavirus disease 2019 (COVID-19) pandemic revealed the vulnerabilities of the FFS model and its reliance on volume-based healthcare delivery. The subsequent decline in patient volumes, with a 19.5% reduction in inpatient and 34.5% reduction in outpatient volumes, led to significant financial losses for hospitals [4]. Between March and June 2020 alone, hospitals reportedly lost $202.6 billion, and projections suggest an additional minimum loss of $120.5 billion from July to December 2020, emphasizing the need for alternative approaches to healthcare delivery [5].

The main disparities in the healthcare system stem from several underlying drivers. Firstly, regulatory bodies often focus on the safety and basic efficacy of new drugs and devices, without necessitating comparative studies against existing treatments. This absence of comparative efficacy requirements allows new therapies to enter the market without proving they offer better outcomes or value. Secondly, the powerful influence of pharmaceutical and medical device companies, through aggressive marketing and lobbying efforts, can sometimes overshadow objective evaluation of the true benefits of a new product. As a result, there is a lack of requirements for new drugs and devices to demonstrate improved effectiveness and cost-effectiveness compared to existing treatments. This environment leads to the adoption of expensive therapies without clear benefits for patients, driving up healthcare costs without necessarily delivering better patient outcomes [6]. Additionally, there is significant variation in prices paid by private insurers, unrelated to the quality of care provided. The delivery of surgical care is often marked by avoidable and costly events, including inappropriate procedures, questionable benefits, and complications resulting in prolonged hospital stays, readmissions, and reinterventions [7]. Value-based care seeks to address these disparities by shifting the focus from quantity of life to quality of life. It emphasizes the need for evidence-based practices, cost-effectiveness, and improved patient outcomes, ensuring that healthcare expenditures align with the value and benefits received by patients [8]. By incentivizing quality, cost-efficiency, and patient-centered care, value-based care aims to reduce disparities and improve the overall effectiveness and value of healthcare delivery [9].

In the context of healthcare, "Value" pertains to the correlation between care quality and the related costs. The Institute of Medicine identifies six crucial domains of value: safety, effectiveness, patient-centeredness, efficiency, timeliness, and equity [10]. These aspects contribute to the overall value patients receive and signify healthcare quality. Value-based care fosters patient outcomes and cost-effectiveness, enabling healthcare providers to deliver efficient, effective, and patient-focused care. It fosters a collaborative and integrated healthcare setting and promotes preventive care, chronic disease management, and healthcare delivery and payment model innovation. Value-based care aims to optimize healthcare delivery, enhancing health outcomes and the overall healthcare service value.

Anesthesiologists play a vital role in actualizing value-based care within the healthcare system. As the healthcare landscape evolves, including a shift towards value-based payment models, anesthesiologists stand in a unique position to impact clinical outcomes and efficiency positively. Their expertise in perioperative care and patient safety allows for active engagement with patients and stakeholders across the surgical care continuum. Their focus on improving clinical outcomes, reducing complications, and optimizing discharge disposition aids in cost avoidance and healthcare delivery efficiency. By aligning their efforts with the goals of value-based care, anesthesiologists can enhance patient outcomes, care coordination, and financial sustainability for healthcare systems.

## Review

Overview of value-based care in anesthesia: applying principles for quality, cost, and patient experience

Value-based care is an approach that focuses on achieving better patient outcomes while optimizing costs. It has gained significant attention in healthcare, including the field of anesthesia, to enhance the quality and efficiency of care delivery. Understanding key concepts of value-based care, such as quality, cost, and patient experience, is essential in applying these principles to anesthesia care [11].

Quality in value-based care encompasses various dimensions, including safety, effectiveness, patient-centeredness, efficiency, timeliness, and equity. Anesthesiologists strive to ensure patient safety by employing evidence-based practices, monitoring vital signs, and managing anesthesia-related risks. Effectiveness is achieved by delivering the right interventions at the right time to achieve optimal outcomes [12]. Patient-centered care involves engaging patients in decision-making, addressing their concerns, and promoting shared decision-making. Efficiency entails maximizing resource utilization while minimizing waste, optimizing workflow, and improving care coordination. Timeliness emphasizes providing timely access to care and minimizing delays. Equity focuses on addressing disparities in healthcare access and outcomes among different patient populations [9].

Cost is a crucial aspect of value-based care, aiming to achieve optimal outcomes at a reasonable expense. Anesthesiologists play a role in cost containment by minimizing unnecessary procedures, optimizing resource utilization, and implementing efficient anesthesia protocols. This includes selecting appropriate medications, optimizing anesthesia techniques, and effectively managing postoperative pain to reduce hospital stays and associated costs [13]. Patient experience is a key component of value-based care, emphasizing the importance of patient satisfaction, engagement, and communication. Anesthesiologists contribute to a positive patient experience by establishing effective communication, addressing patient concerns, and ensuring comfort and safety throughout the perioperative period [8].

While applying value-based care principles to anesthesia care offers opportunities for improving patient outcomes and reducing costs, several challenges exist. Integration of value-based care within anesthesia practice requires a shift from traditional FFS models and adapting to alternative payment models, such as bundled payments or accountable care organizations (ACOs) [14]. Implementing and measuring quality metrics specific to anesthesia care can be complex, requiring standardized data collection and collaboration among stakeholders. Furthermore, addressing the unique needs and variations in surgical procedures, patient populations, and healthcare settings poses challenges in delivering consistent and equitable anesthesia care [4].

Opportunities lie in leveraging technology, such as electronic health records and data analytics, to facilitate data-driven decision-making, optimize care coordination, and measure performance [15]. Collaborating with surgical teams, nurses, and other healthcare professionals enables enhanced care coordination and interdisciplinary communication. Additionally, actively involving patients in the decision-making process and promoting patient education and engagement can improve patient satisfaction and outcomes [16].

To tackle the rising costs of healthcare and substantial operational expenses, FFS models are being replaced with value-based payment methods. Inaccurate billing contributes to approximately 3-10% of total healthcare spending, while administrative costs can account for 20-25% of the nation's healthcare expenditures [17]. To address these issues, healthcare systems are beginning to explore alternative payment models such as bundled reimbursements, accountable care organizations, and penalty-driven programs.

Bundle reimbursements

Under a bundle reimbursement structure, anesthesiologists, surgeons, hospitals, and other care providers involved in a procedure or a course of treatment collaboratively share a single reimbursement. This payment covers the entire spectrum of care, from preoperative evaluations to postoperative follow-ups, and even complications arising within a specified period.

Bundled payments aim to incentivize healthcare providers to deliver efficient, high-quality care, by aligning their financial incentives with patient outcomes. They are designed to eliminate unnecessary services, encourage coordination among care providers, and ultimately reduce healthcare costs while maintaining or improving the quality of care. This payment structure is particularly relevant in the field of anesthesia, where care spans multiple phases of a patient's surgical journey.

In one study, an implementation of this approach was conducted for total joint replacement (TJR) at a large, tertiary, urban academic medical center [18]. The program encapsulated all costs associated with care for up to 90 days post discharge. One year following its introduction, data from 721 Medicare primary TJR patients were analyzed. The findings demonstrated a significant impact on care delivery and cost efficiency. The average hospital stay was reduced from 4.27 days to 3.58 days, with a median length of stay of three days. Furthermore, patient discharges to inpatient facilities saw a considerable decrease from 71% to 44%. Additionally, patient readmissions were slightly reduced post implementation, occurring in 11% of patients (80 individuals). Importantly, the hospital witnessed a reduction in inpatient-related costs over the baseline, supporting the financial efficacy of the program.

While bundled payments hold the potential for improved care coordination and cost efficiency, it's important to recognize the challenges and risks associated with this model [19]. A key concern is the potential for 'under-treatment'. With fixed payments, there is a risk that providers might skimp on services or avoid high-risk patients to minimize costs and maximize profit margins. Moreover, the bundled payment model requires robust data analysis capabilities, meticulous coordination among different care providers, and detailed cost accounting. Such capabilities may be beyond the reach of smaller, resource-limited institutions, leading to potential disparities in care delivery and healthcare outcomes.

ACOs

ACOs represent a transformative shift in the healthcare delivery system, prioritizing a model that enhances care coordination, improves patient outcomes, and reduces healthcare costs [20]. ACOs are groups of physicians, hospitals, and other healthcare providers who voluntarily collaborate to deliver high-quality care to their patients, particularly those enrolled in Medicare. The central premise of an ACO is the commitment to being 'accountable' for the health and wellness of a defined population, striving for improved health outcomes while lowering the cost of care. To achieve these goals, ACOs leverage data-driven decision-making, emphasize preventative care, and focus on managing patients with chronic conditions effectively. Under the ACO model, providers are incentivized to meet specific quality and performance targets, with shared savings programs offering financial rewards for achieving cost and quality benchmarks. This shift from volume to value-based care aims to deliver patient-centric care that enhances patient satisfaction, promotes population health, and manages per capita cost.

One study sought to identify differences in patient outcomes when both hospital and post-acute care (PAC) providers participate in ACOs [21]. By analyzing Medicare claims, the study observed changes in readmission rates, Medicare spending, and length of stay among patients admitted to ACO-participating hospitals and PAC providers. These findings were compared to data from patients discharged from non-ACO-participating facilities over the same timeframe. Patients discharged from ACO‐participating hospitals and skilled nursing facilities (SNF) had lower readmission rates (−1.7 percentage points, p‐value = .03) than before ACO participation and non‐participants; and lower per‐discharge Medicare spending (−$940, p‐value = .001), and length of stay (−3.1 days, p‐value <.001) in skilled nursing facilities.

While ACOs provide certain advantages, one significant concern is the substantial upfront investment required to establish an ACO [22]. Hospitals, physicians, and other healthcare providers must invest in technological infrastructure, such as advanced data analytics and electronic health records, to enable coordinated care and monitor performance metrics. This financial burden can be particularly daunting for smaller or resource-limited providers. Another potential disadvantage is the risk-sharing aspect of ACOs. Although providers have the opportunity to benefit from shared savings if they meet quality and cost targets, they may also face financial penalties if these targets are not met. This potential for financial loss may discourage some providers from participating in ACOs.

Penalty-based model

One popular penalty-based model is the Hospital Readmissions Reduction Program (HRRP). The Hospital Readmissions Reduction Program (HRRP) is a penalty-based initiative under the value-based healthcare umbrella. Introduced by Medicare, the program aims to incentivize hospitals to reduce preventable readmissions [23]. HRRP accomplishes this by reducing Medicare payments to hospitals with high readmission rates for certain conditions, including heart failure, pneumonia, acute myocardial infarction, and hip and knee replacements. The program represents a shift from quantity-focused to quality-focused healthcare, aiming to promote better care coordination, improve patient outcomes, and reduce healthcare costs. The financial penalties imposed by HRRP encourage hospitals to enhance their discharge planning and post-discharge care processes, ultimately reducing the burden of unnecessary hospital readmissions.

One study analyzed the impact of HRRP's expansion to include readmissions following elective primary total hip and knee replacements [24]. By examining Medicare's Hospital Compare datasets from 2009 to 2016, the study compared readmission rates before and after the HRRP expansion. Interestingly, it was found that the HRRP expansion did not lead to greater reductions in postoperative readmissions among hospitals at a higher risk of larger penalties compared to those at risk of smaller penalties. Before the expansion, the average readmission rate was around 5.36-5.46%, and after the expansion, it dropped by nearly 18% (around one percentage point) in all hospitals, regardless of the proportion of their total inpatient revenue attributed to Medicare. This suggests that the HRRP's extension to include joint replacements did not disproportionately affect hospitals based on their reliance on Medicare revenues. The readmission rates were found to be declining at similar rates across all hospitals, irrespective of the HRRP's expansion.

One potential reason for the non-differential impact across hospitals may lie in the complexity of factors contributing to readmissions, which often extend beyond the hospital's control [25]. Social determinants of health, such as patient lifestyle, socioeconomic status, and adherence to postoperative care instructions, play a crucial role in readmissions and are difficult to address within the hospital setting. Furthermore, the penalties associated with the HRRP, while intended to drive behavior change, may not have been sufficiently large or well-targeted to create a significant differential impact across hospitals. Future value-based models may need to consider more nuanced incentive structures or provide additional resources to effectively address the diverse and complex factors influencing readmissions. Despite the mixed evidence with various value-based payment models, evidence has shown that multidisciplinary teams can make an impactable difference when it comes to perioperative and postoperative care.

With a variety of payment models, each has its advantages and disadvantages as shown in Table 1. Regardless of the type of model used, anesthesiologists are making advances in their perioperative and postoperative care in order to provide the most value to patients in accordance with the payment models. 

**Table 1:** Summary of Various Value-Based Care Models

Model	Key Features	Focus Area	Advantage	Challenges/Outcomes
Bundle Reimbursements	- Collaborative sharing of reimbursement - Covers preoperative to postoperative care	Procedure/course of treatment	Incentivize efficient, high-quality care	- Potential for under-treatment: Providers may skimp on services or avoid high-risk patients to minimize costs and maximize profit margins - Data analysis and coordination challenges: Robust data analysis and coordination among care providers are required - Resource limitations: Smaller institutions may face challenges due to financial constraints and lack of infrastructure
Accountable Care Organizations (ACOs)	- Collaboration among healthcare providers -Data-driven decision-making and quality targets	Coordinated care	Improved patient outcomes, shared savings programs	- Upfront investment: Establishing ACOs requires investment in infrastructure - Risk-sharing and financial penalties: Providers may face penalties for not meeting quality and cost targets - Participation barriers: Smaller providers may find it challenging to meet participation requirements
Penalty-Based Model	- Financial penalties for high readmission rates - Incentivizes better discharge planning and care	Reduce readmissions	Improve care coordination, reduce unnecessary readmissions	- Complex factors beyond hospital control can influence readmission rates - Potential for insufficient penalties: Financial penalties may not be large enough to drive significant improvement - Disparities in impact: The model's impact may vary across hospitals

Perioperative care

As per the data from the National Council on Aging (2021), around 80% of older adults in the United States are living with a minimum of one chronic disease, while 77% are managing at least two [26]. The high risk of postoperative complications for surgical patients with multiple chronic conditions necessitates an increasing focus on effective and safe perioperative care, especially considering the growing populations with complex chronic diseases and older adults. The current average cost per instance of a surgical complication is around $19,000, a figure that underscores the potential for spiraling healthcare costs if this trend remains unchecked [27]. As it stands, an estimated 18.3% of the United States gross domestic product is already committed to healthcare costs [28]. These factors collectively emphasize the imperative for health professionals, health systems, payers, and patients to prioritize managing this pressing issue.

Duke University has instituted a Perioperative Enhancement Team (POET) to enhance the perioperative care process [11]. The central assembly of the POET comprises medical professionals from an array of specializations, including anesthesiology, surgery, and internal medicine. Duke further stratified this strategy into a preoperative anemia clinic, a preoperative diabetes clinic, a preoperative nutrition optimization clinic, and a perioperative pain clinic.

The idea of a perioperative pain clinic represents a unique opportunity for pain management anesthesiologists. A recent study showed that patients with a diagnosis of opioid dependence had a higher 30-day readmission rate, longer mean length of hospital stay, and higher estimated hospital costs [29]. The goal of the clinic is to preoperatively minimize the risk of postsurgical pain and to address limiting pain and biosocial factors [30]. In particular, the management of chronic opioid users presents a distinct challenge in perioperative care. These patients often experience more intense and longer-lasting postoperative pain, resulting in poorer outcomes and increased healthcare costs. Anesthesiologists, with their proficiency in pain management, can contribute significantly to creating personalized care plans for these patients. These plans can incorporate various strategies including opioid-sparing techniques, regional anesthesia, and utilization of multimodal analgesia, thereby improving pain control, and potentially reducing the need for postoperative opioids.

Furthermore, given the increasingly prevalent role of technology and artificial intelligence in the healthcare landscape, the use of machine learning algorithms for risk quantification can further aid medical professionals in preoperative care. The University of Pittsburg implemented a risk analysis index (RAI) to predict post-surgical outcomes [31]. Notably, the RAI score showed a significant association with the risk of death and was used as a diagnostic test for frailty by calculating the sensitivity, specificity, positive predictive value (PPV), and negative predictive value (NPV) at a certain RAI threshold for various clinical outcomes. The potential utility of the RAI extends to the postoperative phase as well. Patients identified as high-risk based on their RAI score might benefit from a more tailored, proactive postoperative pain management plan, implemented by the anesthesiologist in collaboration with the surgical team. This can involve a multimodal analgesic approach, incorporating various types of pain medication to minimize opioid use and side effects. It might also involve coordinating with physical therapists, nutritionists, and other providers to address the full spectrum of factors that contribute to recovery and long-term outcomes.

Postoperative care

Enhanced recovery after surgery (ERAS) represents a paradigm shift in perioperative care and is a vital aspect of value-based healthcare delivery. ERAS pathways integrate evidence-based practices across the entire surgical journey, encompassing preoperative preparation, intraoperative management, and postoperative recovery. These principles are designed to optimize patient outcomes, improve recovery times, and reduce the length of hospital stays, which aligns directly with the objectives of value-based care, aiming to maximize health outcomes per unit of cost [32].

Anesthesiologists, as central figures in the perioperative team, play an essential role in the implementation of ERAS protocols. Their expertise extends beyond intraoperative anesthetic management, to preoperative optimization and postoperative pain and symptom management. This holistic involvement allows anesthesiologists to have a significant impact on patient recovery trajectories.

A recent study looked at 53 patients undergoing cardiac surgery before the implementation of an enhanced recovery after cardiac surgery (ERACS) protocol (pre-ERACS group) and 52 patients undergoing cardiac surgery after the implementation of an ERACS protocol (ERACS group) [33]. Patients in the ERACS group were given detailed preoperative information, avoidance of prolonged fasting periods preoperatively, preoperative carbohydrate beverages, optimization of analgesia with avoidance of long-acting opioids, prevention of postoperative nausea and vomiting, early enteral nutrition postoperatively, and early mobilization. The results of the study showed that there was a statistically significant reduction in the number of patients in the ERACS group presenting with one or more postoperative complications (including hospital-acquired infections, acute kidney injury, atrial fibrillation, respiratory failure, postoperative myocardial infarction, and death). In addition, postoperative pain scores were improved significantly in the ERACS group.

Another application of this idea has been in the use of transcatheter aortic valve replacement (TAVR) surgeries at Houston Methodist Hospital [32]. Initially, the focus of this program was on refining anesthetic management, but it quickly expanded to cover all aspects of perioperative care. As expertise in performing TAVR procedures increased, the use of general endotracheal anesthesia (GETA) was replaced with conscious sedation, achieved through the administration of propofol and dexmedetomidine infusions. In order to reduce the need for narcotics and the associated side effects, the surgical site was anesthetized locally before the incision, supplemented by intravenous acetaminophen for pain management. Central venous catheters utilized during the procedure began to be routinely removed in the operating room. Instead of transesophageal echocardiography (TEE), transthoracic echocardiography was employed to evaluate the success of the surgical repair and to guide fluid and inotropic therapy. The procedural time was effectively reduced, leading to a diminished need for routine urinary catheter placement, subsequently lowering the risk of catheter-associated urinary tract infections (CAUTIs). Unless there was an occurrence of severe bradycardia or a new bundle branch block, the temporary pacemaker lead, placed via the central venous catheter, was typically removed. Following the procedure, patients were transferred to the post-anesthesia care unit (PACU) and subsequently to the cardiac ward, which resulted in decreased ICU utilization. For a quicker recovery, patients were mobilized just hours after arrival in the PACU.

As healthcare systems continue to transition from FFS models to value-based care, the role of anesthesiologists in implementing and managing ERAS protocols is poised to become increasingly vital. Their contributions extend the value of anesthesiology beyond the operating room and highlight the discipline's significance in holistic, patient-centered care.

## Conclusions

Anesthesiologists have a unique opportunity to drive the advancement of value-based care, working collaboratively with healthcare stakeholders to improve patient outcomes, standardize practices, and optimize resource utilization. By embracing their role as leaders, leveraging technology and data, and engaging in research, anesthesia providers can make significant contributions to delivering high-value care that benefits patients, hospitals, and the healthcare system. This review highlights the valuable role anesthesiologists can play in advancing value-based care in surgical settings. Perioperative medicine is not a departure from their current roles but a rediscovery of their significance in the healthcare community. The review emphasizes the need for collaboration with hospital and surgeon leadership, the establishment of clinical pathways and quality metrics, and the utilization of data and clinical decision support for continuous improvement. The main findings emphasize the importance of increasing value, standardizing practices, and sharing results with other institutions for collective improvement.

Further research is needed to explore the integration of technology, such as electronic health records and anesthesia information management systems, in optimizing anesthesia care quality, efficiency, and cost-effectiveness. Additionally, investigating the impact of perioperative interventions, such as enhanced recovery protocols and opioid-sparing analgesia, on patient outcomes and healthcare costs would provide valuable insights for future practice.
